# Implication of overexpression of dishevelled-associated activator of morphogenesis 1 (Daam-1) for the pathogenesis of human Idiopathic Pulmonary Arterial Hypertension (IPAH)

**DOI:** 10.1186/s13000-017-0614-7

**Published:** 2017-03-14

**Authors:** Shun Yanai, Megumi Wakayama, Haruo Nakayama, Minoru Shinozaki, Hisayuki Tsukuma, Naobumi Tochigi, Tetsuo Nemoto, Tsutomu Saji, Kazutoshi Shibuya

**Affiliations:** 1 Department of Pediatrics, Toho University School of Medicine, 6-11-1 Omori-nishi, Ota-ku, Tokyo 143-8541 Japan; 2 Department of Surgical Pathology, Toho University School of Medicine, 6-11-1 Omori-nishi, Ota-ku, Tokyo 143-8541 Japan; 3 Department of Neurosurgery, Toho University Ohashi Medical Center, 2-17-6 Ohashi, Meguro-ku, Tokyo 153-8515 Japan; 4 Toho University School of Medicine, 5-21-16 Omori-nishi, Ota-ku, Tokyo 143-8540 Japan; 5 Advanced and Integrated Cardiovascular Research Course in the Young and Adolescence, Toho University School of Medicine, 5-21-16 Omori-nishi, Ota-ku, Tokyo 143-8540 Japan

**Keywords:** IPAH, Wnt/PCP pathway, Daam-1, Smooth muscle cells, Overexpression

## Abstract

**Background:**

Idiopathic pulmonary arterial hypertension (IPAH) is a rare, fatal disease of unknown pathogenesis. Evidence from our recent study suggests that IPAH pathogenesis is related to upregulation of the Wnt/planar cell polarity (Wnt/PCP) pathway. We used microscopic observation and immunohistochemical techniques to identify expression patterns of cascading proteins—namely Wnt-11, dishevelled-2 (Dvl-2), and dishevelled-associated activator of morphogenesis 1 (Daam-1)—in pulmonary arteries.

**Methods:**

We analyzed sections of formalin-fixed and paraffin-embedded autopsied lung tissues obtained from 9 IPAH cases, 7 associated pulmonary arterial hypertension cases, and 16 age-matched controls without pulmonary arterial abnormalities. Results of microscopic observation were analyzed in relation to the cellular components and size of pulmonary arteries.

**Results:**

Varying rates of positive reactivity to Dvl-2 and Daam-1 were confirmed in all cellular components of pulmonary arteries, namely, endothelial cells, myofibroblasts, and medial smooth muscle cells. In contrast, none of these components was reactive to Wnt-11. No specific expression patterns were observed for endothelial cells or myofibroblasts under any experimental conditions. However, marked expression of Dvl-2 and Daam-1 was confirmed in smooth muscle cells. In addition, Dvl-2 was depleted while Daam-1 expression was elevated in IPAH, in contrast with specimens from associated pulmonary arterial hypertension cases and controls.

**Conclusions:**

High Daam-1 expression may upregulate the Wnt/PCP pathway and cause IPAH.

## Background

Although the first autopsy case was described by Romberg in 1891, the cause of idiopathic pulmonary arterial hypertension (IPAH) is unknown [1]. Prognosis has been poor because of the eventual development of right heart failure caused by progression of pulmonary vascular resistance [2]. The recent development of disease-modifying drugs such as the prostaglandin I_2_ analogs, endothelin receptor antagonists, and phosphodiesterase type-5 inhibitors has dramatically improved outcomes for patients with pulmonary arterial hypertension (PAH); however, most patients still develop chronic right heart failure, for which the only curative treatment is lung transplantation [3].

During the past decade, several studies have investigated the pathogenesis of IPAH, particularly mutations in bone morphogenetic protein receptor II (BMPR2), a transforming growth factor β (TGFβ) superfamily receptor. *BMPR2* mutations are present in 80% of patients with heritable pulmonary arterial hypertension, a subgroup of IPAH patients with familial accumulation [4]. However, only 20% of *BMPR2*-positive individuals develop IPAH during their lifetime and the male:female ratio of patients is 1:2.5. These findings complicate IPAH pathogenesis, and progress in treatment has thus been slow [5, 6].

We fortuitously discovered that *Stachybotrys chartarum*, a ubiquitous fungus in our environment, induces PAH in mice, which was confirmed physiologically and histologically [7]. Previously reported animal models induced IPAH by monocrotaline injection or by breeding of animals in a hypoxic environment; however, these models may not reflect the pathophysiology of human PAH [8]. Results of RNA microarray assay analysis indicated that, as compared with these conventional models, pulmonary arterial lesions from our model exhibited gene expression patterns that were more similar to those of human IPAH [9]. Fluctuations in signal transduction pathways were compared with those reported in 2009 for humans, and upregulation of the Wnt/Planar cell polarity (Wnt/PCP) pathway was implicated in human IPAH pathogenesis [10]. To confirm the mechanism responsible for upregulation of the Wnt/PCP pathway, we used immunohistochemical (IHC) techniques to investigate expression and localization of important Wnt/PCP pathway cascading proteins in human pulmonary arteries (PA) obtained from autopsy cases.

## Methods

### Subjects

All autopsy cases with a diagnosis of PAH (IPAH or APAH) recorded at Toho University Omori Medical Center during the period from 1958 to 2011 were included in this study. Age-matched controls without pulmonary arterial abnormalities were selected separately. This study was approved by the Ethics Committee of Toho University School of Medicine (approval no. 2709425035).

### Selection of tissues for built-in/controls

Built-in controls for Dvl-2 and Daam-1 were set in bronchial epithelium. The control for Wnt-11 was set in the gastric fundic gland, as indicated in The Human Protein Atlas [11].

### Preparation of samples: conventional staining

Formalin-fixed and paraffin-embedded (FFPE) tissues of lungs from autopsy subjects were cut into sections (thickness, 3 μm) and mounted on slide glasses. After deparaffinization, samples were stained with hematoxylin and eosin and Elastica van Gieson. FFPE stomach tissues from 2 selected IPAH patients were prepared in the same manner.

### Preparation of samples: IHC

FFPE lung and stomach tissues were cut into sections (thickness, 3 μm). After deparaffinization, samples were immersed in 0.1% trypsin solution and heated to 95 °C by water bath, for antigen retrieval. Staining was done with the universal immunoenzyme polymer method, a double staining method developed by Nichirei Biosciences, Tokyo, Japan [12]. After blocking with 3% peroxidase methanol for 10 min, 2 drops of the primary antibody were added for 30 min. The samples were then washed and Simple Stain MAX-PO MULTI (Nichirei Biosciences, Tokyo, Japan) was added as a common secondary antibody for another 30 min. Anti–Wnt-11 antibody (dilution 1:50, Atlas Antibodies, Stockholm, Sweden), anti–Dvl-2 antibody (dilution 1:50, Santa Cruz Biotechnology, Dallas, TX, USA), and anti–Daam-1 antibody (dilution 1:200, Proteintech, Chicago, IL, USA) were used as primary antibodies. After color development with diaminobenzidine, samples were stained with hematoxylin.

### IHC analysis

During optical microscopic observation, the vessel diameter of PA was classified into 2 categories, according to Brenner’s classification of pulmonary arteries and arterioles defined at a diameter of 100 μm [13]. Cellular components of the pulmonary arterial walls were classified into 3 categories: endothelial cells, myofibroblasts (for PAH cases), and smooth muscle cells (SMC). All slides were evaluated by 3 independent, board-certified pathologists.

### Statistical analyses

The rates of positive expression of Dvl-2 and Daam-1 were evaluated as a single trend, to identify expression patterns. Thus, the comparison of 2-means method was used to compare the odds ratios for positive expression rates of Dvl-2 and Daam-1 between groups. In this analysis, control and IPAH specimens, and control and APAH specimens, were compared separately.

## Results

### Clinical data

The salient characteristics of the autopsy cases are shown in Table 1.

**Table 1:** Characteristics of autopsy cases

A. PAH Cases					
Patient	Diagnosis	H-E Grade	Age (years)	Sex	Underlying conditions
1^a^	APAH	II	4 months	Female	DORV and VSD
2^a^	APAH	II	5 months	Female	Trisomy 18 and VSD
3^a^	APAH	II	6 months	Female	DORV and VSD
4^a^	APAH	II	7 months	Female	Trisomy 21, ASD, and VSD
5	IPAH	III	13	Female	
6	IPAH	IV	16	Female	
7^a^	IPAH	IV	17	Female	
8^a^	IPAH	IV	19	Female	
9^a^	IPAH	III	21	Female	
10	IPAH	IV	24	Male	
11	APAH	VI	29	Male	Collagen disease
12	IPAH	IV	39	Female	
13	APAH	IV	41	Male	Malignant lymphoma
14^a^	IPAH	III	44	Female	
15^a^	IPAH	IV	51	Female	
16^a^	APAH	III	63	Male	Collagen disease
B. Age-matched controls					
Patient	Age (years)	Sex	Underlying conditions		
17	3	Female	Pneumonia		
18^a^	4	Male	Leukemia		
19^a^	11	Female	Biliary atresia		
20	14	Female	Leukemia		
21^a^	14	Male	Leukemia		
22^a^	15	Male	Leukemia		
23^a^	16	Male	Hypertrophic cardiomyopathy		
24	17	Male	Idiopathic myocarditis		
25^a^	18	Male	Ewing sarcoma		
26^a^	22	Female	Suicide		
27^a^	23	Female	Cerebral hemorrhage		
28^a^	29	Male	Chronic hepatitis		
29	42	Male	Anterior mediastinal tumor		
30	43	Female	Breast cancer		
31^a^	47	Female	Gastric cancer		
32^a^	56	Female	Subacute hepatitis		
C. Summary					
	No.	Median age	Males:females		
PAH cases	16	20 y (4 m - 63 y)	4:12		
IPAH	9	21 y (13 y - 51 y)	1:8		
APAH	7	7 m (4 m - 63 y)	3:4		
Age-matched controls	16	18 y (3 y - 56 y)	8:8		

Cases are in ascending order of age

H-E Grade, Heath–Edwards grade ^36^; DORV, double outlet right ventricle; VSD, ventricular septal defect; ASD, atrial septal defect

^a^ indicates that positive reactivity against both anti–Dvl-2 antibody and anti–Daam-1 antibody was confirmed in built-in control before further evaluation

### IHC of built-in/controls

Wnt-11 was expressed in 100% (2/2) of control samples. The positive expression rate of Dvl-2 was 56% (5/9) for IPAH cases, 71% (5/7) for APAH cases, and 69% (11/16) for age-matched controls. The positive expression rate for Daam-1 was 100% (9/9 and 7/7) for IPAH and APAH cases and 94% (15/16) for age-matched controls.

### IHC of pulmonary arteries (Figs. 1, 2, 3, 4 and 5)

**Fig. 1:**
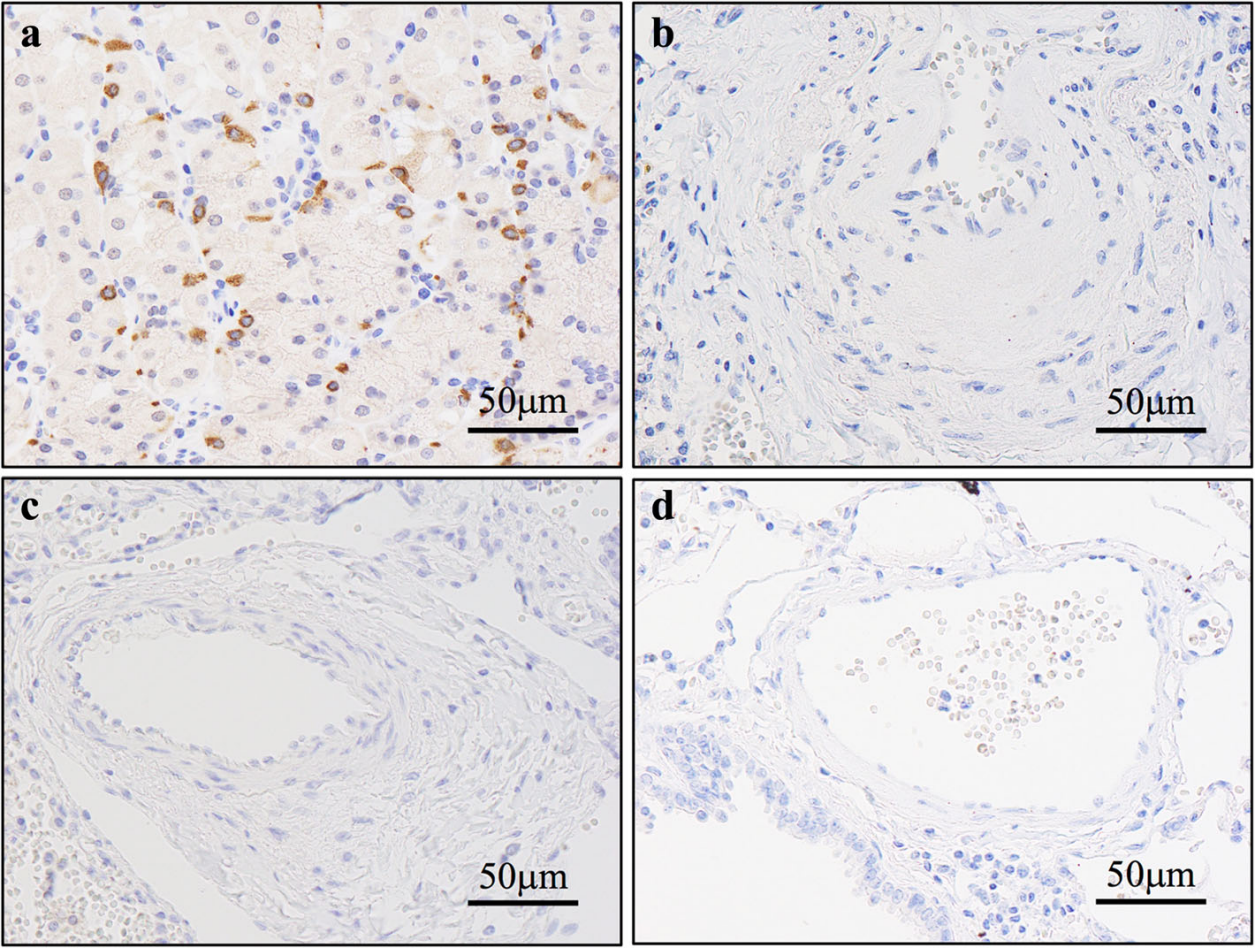
Photomicrographs of anti–Wnt-11 antibody immunostaining. **a** Control: positive reactivity is confirmed in parietal cells of gastric fundic glands. **b** IPAH, PA: positive reactivity is not confirmed in endothelial cells, myofibroblasts, or SMC. **c** APAH, PA: positive reactivity is not confirmed in endothelial cells, myofibroblasts, or SMC. **d** Age-matched control, PA: positive reactivity is not confirmed in endothelial cells, myofibroblasts, or SMC

**Fig. 2:**
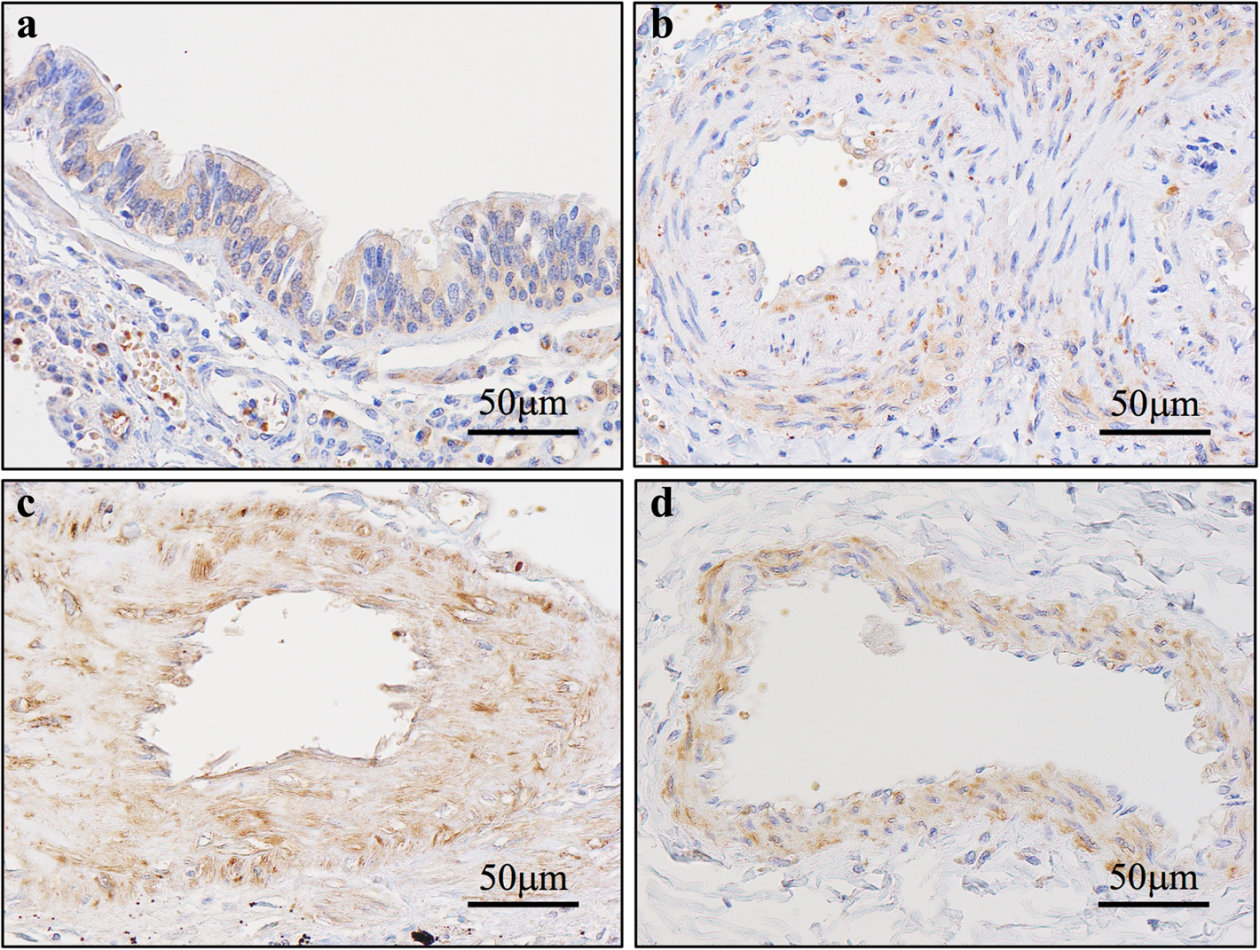
Photomicrographs of anti–Dvl-2 antibody immunostaining. **a** Built-in control: positive reactivity is confirmed in bronchial epithelial cells. **b** IPAH, PA: positive reactivity is confirmed in endothelial cells and SMC. **c** APAH, PA: positive reactivity is confirmed in endothelial cells and SMC. **d** Age-matched control, PA: positive reactivity is confirmed in endothelial cells and SMC

**Fig. 3:**
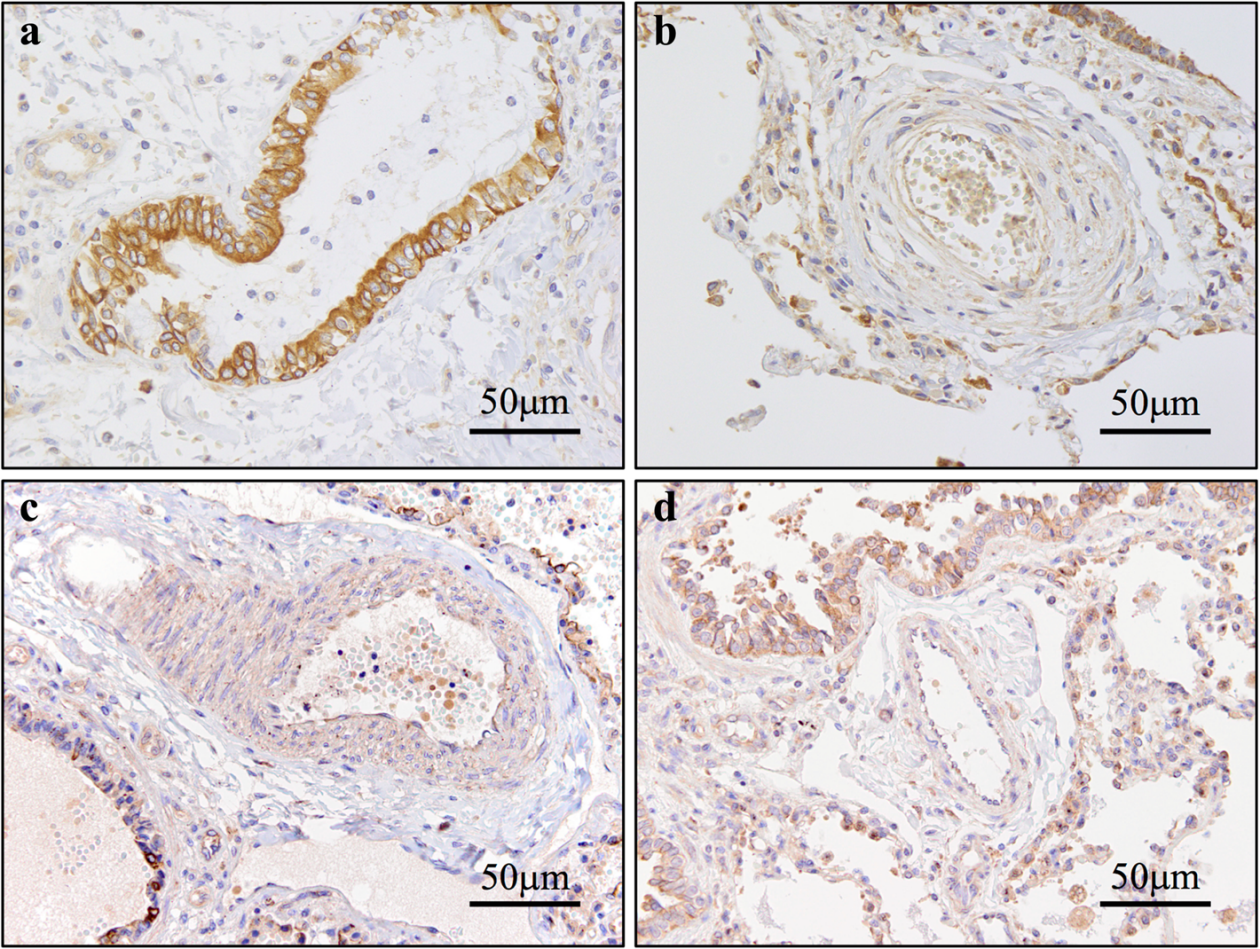
Photomicrographs of anti–Daam-1 antibody immunostaining. **a** Built-in control: positive reactivity is confirmed in bronchial epithelial cells. **b** IPAH, PA: positive reactivity is confirmed in endothelial cells, and SMC. **c** APAH, PA: positive reactivity is observed in endothelial cells and SMC. **d** Age-matched control, PA: positive reactivity is observed in endothelial cells and SMC

**Fig. 4:**
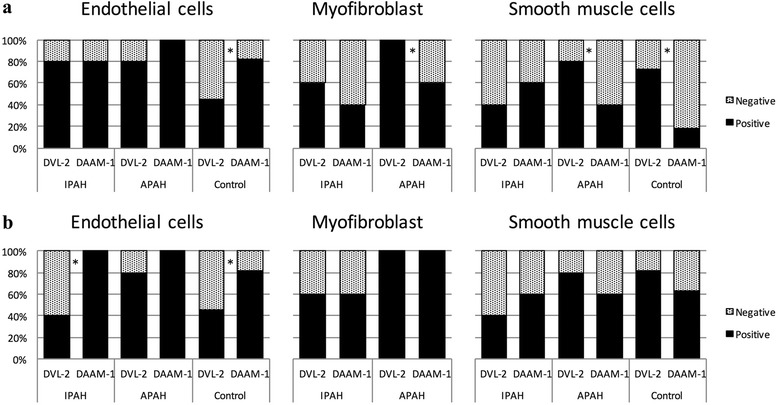
Positive expression rates of Dvl-2 and Daam-1 in pulmonary arteries from autopsy cases. **a**, Small arteries. **b**, Medium-sized arteries. * *P* < 0.05

**Fig. 5:**
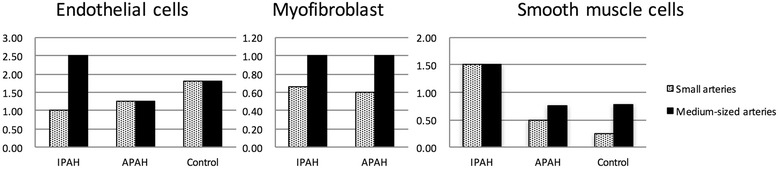
Ratio of positive expression rates (Daam-1/Dvl-2)

Because of the low positive expression rate for Dvl-2 for the built-in controls, pulmonary arteries were evaluated only for built-in control samples that were positive for both Dvl-2 and Daam-1.

#### Endothelial cells

##### Small arteries

No Wnt-11–positive cells were observed under any experimental conditions. The positive expression rates for Dvl-2 and Daam-1 were 80% (4/5) and 80% (4/5), respectively, for IPAH cases, 80% (4/5) and 100% (5/5) for APAH cases, and 45% (5/11) and 82% (9/11) for the controls.

##### Medium-sized arteries

No Wnt-11–positive cells were observed under any experimental conditions. The positive expression rates for Dvl-2 and Daam-1 were 40% (2/5) and 100% (5/5), respectively, for IPAH cases, 80% (4/5) and 100% (5/5) for APAH cases, and 45% (5/11) and 82% (9/11) for controls.

#### Myofibroblasts

##### Small arteries

No Wnt-11–positive cells were observed under any experimental conditions. Positive expression rates for Dvl-2 and Daam-1 were 60% (3/5) and 40% (2/5), respectively, for IPAH cases and 100% (5/5) and 60% (3/5) for APAH cases.

##### Medium-sized arteries

No Wnt-11–positive cells were observed under any experimental conditions. Positive expression rates for Dvl-2 and Daam-1 were 60% (3/5) and 60% (3/5), respectively, for IPAH cases and 100% (5/5) and 100% (5/5) for APAH cases.

#### SMC

##### Small arteries

No Wnt-11–positive cells were observed under any experimental conditions. Positive expression rates for Dvl-2 and Daam-1 were 40% (2/5) and 60% (3/5), respectively, for IPAH cases, 80% (4/5) and 40% (2/5) for APAH cases, and 73% (8/11) and 18% (2/11) for controls.

##### Medium-sized arteries

No Wnt-11–positive cells were observed under any experimental conditions. Positive expression rates for Dvl-2 and Daam-1 were 40% (2/5) and 60% (3/5), respectively, for IPAH cases, 80% (4/5) and 60% (3/5) for APAH cases, and 82% (9/11) and 64% (7/11) for controls.

### Statistical analysis: comparison of 2-means (Table 2)

**Table 2:** Comparison by 2-means test

Small arteries		Positive cases (n)	Negative cases (n)	Odds ratio	Log odds ratio	Approximate variance	z value	P value
A. Endothelial cells								
Control	Dvl-2	5	6					
	Daam-1	9	2	0.19	-1.69	0.98		
IPAH	Dvl-2	4	1					
	Daam-1	4	1	1.00	0.00	2.50	-0.90	0.37
APAH	Dvl-2	4	1					
	Daam-1	5	0.5	0.40	-0.92	3.45	-0.37	0.71
Medium-sized arteries								
Control	Dvl-2	5	6					
	Daam-1	9	2	0.19	-1.69	0.98		
IPAH	Dvl-2	2	3					
	Daam-1	5	0.5	0.07	-2.71	3.03	0.51	0.61
APAH	Dvl-2	4	1					
	Daam-1	5	0.5	0.40	-0.92	3.45	-0.37	0.71
B. Myofibroblasts								
IPAH	Dvl-2	3	2					
	Daam-1	2	3	2.25	0.81	1.67		
APAH	Dvl-2	5	0.5					
	Daam-1	3	2	6.67	1.90	3.03	-0.50	0.62
Medium-sized arteries								
IPAH	Dvl-2	3	2					
	Daam-1	3	2	1.00	0.00	1.67		
APAH	Dvl-2	5	0.5					
	Daam-1	5	0.5	1.00	0.00	4.40	0.00	1.00
Smooth muscle cells								
Control	Dvl-2	8	3					
	Daam-1	2	9	12.00	2.48	1.07		
IPAH	Dvl-2	2	3					
	Daam-1	3	2	0.44	-0.81	1.67	1.99	0.05
APAH	Dvl-2	4	1					
	Daam-1	2	3	6.00	1.79	2.08	0.39	0.70
Medium-sized arteries								
Control	Dvl-2	9	2					
	Daam-1	7	4	2.57	0.94	1.00		
IPAH	Dvl-2	2	3					
	Daam-1	3	2	0.44	-0.81	1.67	1.07	0.28
APAH	Dvl-2	4	1					
	Daam-1	3	2	2.67	0.98	2.08	-0.02	0.98

The odds ratios for positive expression rates of Dvl-2 and Daam-1 were compared between control and IPAH specimens and between control and APAH specimens. A, endothelial cells. B, myofibroblasts. C, smooth muscle cells

#### Endothelial cells

##### Small arteries

The P-values for the difference in odds ratios were 0.37 for IPAH specimens versus controls and 0.71 for APAH specimens versus controls.

##### Medium-sized arteries

The P-values for the difference in odds ratios were 0.61 for IPAH specimens versus controls and 0.71 for APAH specimens versus controls.

#### Myofibroblasts

##### Small arteries

The P-value for the difference in odds ratios was 0.62 for IPAH versus APAH specimens.

##### Medium-sized arteries

The P-value for the difference in odds ratios was 1.00 for IPAH versus APAH specimens.

#### SMC

##### Small arteries

The P-values for the difference in odds ratios were 0.05 for IPAH specimens versus control and 0.70 for APAH specimens versus controls.

##### Medium-sized arteries

The P-values for the difference in odds ratios were 0.28 for IPAH specimens versus control and 0.98 for APAH specimens versus controls.

## Discussion

### Genetics and pathogenesis of IPAH

Although more than 90% of IPAH cases are sporadic, familial accumulation of IPAH has long been recognized [14]. Such cases were classified as familial pulmonary (arterial) hypertension (FPAH) as early as 1973 [15]. However, after serial reports of mutations in *BMPR2* and other TGFβ-related genes in FPAH patients in the early 2000s [5, 6, 16], the term HPAH replaced FPAH in the Dana Point classification of 2009. HPAH is used to refer to patients with newly diagnosed IPAH and genetic mutations and those previously classified as having FPAH [17]. According to the Nice classification of 2013, which succeeded the Dana Point classification, up to 80% of HPAH patients present with *BMPR2* mutations, and an additional 5% present with mutations in other TGFβ superfamily genes, such as activin receptor-like kinase 1 (*ALK1*), endoglin (*ENG*), *SMAD4*, *SMAD8,* and caveolin1 (*CAV1*) [5]. Among TGFβ superfamily receptors, the TGF receptors are believed to promote proliferation and maturation of pulmonary arterial SMC (PASMC), whereas BMP receptors appear to suppress proliferation of PASMC and apoptosis of arterial endothelial cells [18, 19]. Thus, a mutation in any TGFβ superfamily receptor gene could trigger an imbalance between TGF and BMP receptors, thereby leading to aberrant contraction and proliferation of PASMC and, ultimately, IPAH [20, 21].

Several studies using animal models have reported interaction of BMPR2 and TGFβ superfamily receptors, which supports the hypothesis of TGF–BMP imbalance [22–24]. One report showed depletion of BMPR2 with sustained expression of TGFβ2 receptors in human tissues [23]. However, *BMPR2* mutation is present in only 10% to 40% of IPAH patients, and only 20% of individuals with a *BMPR2* mutation develop IPAH during their lifetime [5, 6, 16]. These findings regarding *BMPR2* mutations in IPAH patients and development of IPAH among individuals with *BMPR2* mutations suggest that unrevealed signal transduction pathways are responsible for IPAH pathogenesis, either in cooperation with, or independent of, dysfunction in TGFβ systems [9].

A 2008 study confirmed the high reproducibility of pulmonary arterial remodeling and elevated systolic pressure in the right ventricle, which closely mimics IPAH, after repeated intratracheal injection of *S. chartarum* in otherwise normal male ddY mice [7]. This fungus species is ubiquitous in our environment, and very few cases of *S. chartarum* infection have been reported [7, 8, 25–28].

A 2012 study reported the results of molecular biological analyses of a mouse model of IPAH induced by repeated intratracheal injection of *S. chartarum*. In that study, lung RNA expression profiles of the PAH model mice were assayed with a DNA microarray technique for gene ontology and pathway analyses [9]. Candidate signal transduction pathways were then compared with the results of a DNA microarray assay analysis of human IPAH, which were published in 2009 [10]. The signal expression patterns of the mouse PAH model and human IPAH were very similar. Commonalities in fluctuations in signal transduction pathways in human IPAH and the mouse PAH model were confirmed in the following pathways: upregulation of Janus kinase/signal transducers and activators of the transcription (JAK/STAT) pathway, the hemostasis pathway, the estrogen receptor pathway, and the serotonin receptor pathway, and down-regulation of the vascular endothelial growth factor (VEGF) pathway, the platelet-derived growth factor (PDGF) pathway, apoptosis, and the BMP signaling pathway. Because of the marked similarities in signal expression patterns between human IPAH and our PAH model animal prepared by *S. chartarum*, there were only 3 signal transduction pathways extracted that were uniquely affected in human IPAH PA in higher hierarchical pathways, namely, upregulation of the Wnt/PCP pathway and the succeeding Ras homolog gene family, member A/Rho-associated, coiled-coil–containing protein kinase (RhoA/ROCK) pathway, and down-regulation of the TGFβ pathway. The RhoA/ROCK pathway induces contraction and proliferation of medial SMC [29, 30] and is positively regulated by the Wnt/PCP and TGFβ pathways. This suggests that upregulation of the Wnt/PCP pathway leads to subsequent upregulation of the RhoA/ROCK pathway and that down-regulation of TGFβ pathway is affected by down-regulation of its higher hierarchical pathway or by negative feedback from the RhoA/ROCK pathway [31, 32]. Recent studies of fasudil, a RhoA/ROCK inhibitor, also indicate that the RhoA/ROCK pathway is an important factor in IPAH development, as IPAH improved after fasudil was given to experimental animals and human IPAH patients [33–35].

### IHC analysis of Wnt/PCP pathway cascading proteins in human PA

The present study aimed to directly confirm expression and localization of major cascading proteins of Wnt/PCP pathway, namely Wnt-11, Dvl-2, and Daam-1, on pulmonary arterial walls. Because biopsy specimens of pulmonary arteries are not easy to obtain, all samples were obtained in the form of FFPE from autopsy, and IHC was chosen as a simple means of examination. Because of the unstable nature of IHC, we used a painstaking 2-step procedure to confirm our results for Dvl-2 and Daam-1. In the first step, a built-in control was observed for reactivity with the primary antibody, and slides were selected for further evaluation only when positive reactivity was confirmed for the built-in control.

Because Wnt-11 was not observed in any PA specimen but was confirmed in parietal cells of gastric fundic glands, our evaluation focused on the expression patterns for Dvl-2 and Daam-1. Observation of endothelial cells and myofibroblasts showed no characteristic expression patterns for Dvl-2 and Daam-1 under any experimental conditions. However, in SMC, there was a clear difference in expression patterns between experimental conditions. Whereas the positive expression rate for Daam-1 was lower than that for Dvl-2 in control specimens without pulmonary arterial abnormalities and in APAH specimens, the positive expression rate for Daam-1 was higher than that for Dvl-2 in IPAH specimens (Fig. 4). In other words, the expression pattern for IPAH contrasted with those for APAH and control specimens. When the ratio of positive expression rates was compared in SMC, the Daam-1/Dvl-2 ratio was 1.5 for small and medium-sized arteries in IPAH but less than 1.0 for control and APAH, and the value was even lower for small arteries (Fig. 5).

To confirm the specificity of signal expression patterns, we used the 2-means method to compare the odds ratios of the positive expression rates for Dvl-2 and Daam-1. We found that in small arteries the expression patterns for Dvl-2 and Daam-1 in IPAH PASMC differed from those for control PASMC (P = 0.05), whereas the difference between APAH PASMC and control PASMC was not significant (P = 0.70). For medium-sized arteries, the P-value was 0.28 for the comparison between IPAH PASMC and control PASMC and 0.98 for the comparison between APAH PASMC and control PASMC. Although both differences were nonsignificant, the lower P-value for the former might reflect the particular characteristics of the expression patterns of Dvl-2 and Daam-1 in IPAH PASMC.

The results showed a greater difference in Dvl-2/Daam-1 expression rate for small arteries than for medium-sized arteries. This tendency reflects the accepted characteristics of IPAH progression, namely, small arteries are affected first and medium-sized arteries are affected after substantial progression of lesions in small arteries [2, 4, 13, 36].

### Wnt/PCP pathway

Wnt/PCP pathway is 1 of the 3 known Wnt family signal transduction pathways related to cell migration and polarity that are conserved across most vertebrates [37]. First discovered in 1980s, this pathway was initially studied by researchers in embryogenesis, then by researchers in oncology [38, 39]. Studies of embryogenesis suggest that the Wnt/PCP pathway is responsible for cardiogenesis and vasculogenesis [40, 41].

The Wnt/PCP signal transduction pathway is initiated by conjugation of the Wnt ligand to the 7-transmembrane Fz receptor, where it activates Dvl-2 in cytosol by an unknown mechanism. Activated Dvl-2 conjugates with Daam-1, which is autoinhibited in its natural state by its characteristic conformation and requires transformation into a binary formation with Dvl-2 for active function. Activated Daam-1 subsequently activates the RhoA/ROCK pathway, which leads to continuous medial SMC contraction and proliferation [37, 42].

The Kyoto Encyclopedia of Genes and Genomes [43] and the Reactome Pathway Database [44] indicate that the Wnt/PCP pathway reserves no known alternate or collateral pathways that complement pathway function in cases of impaired expression or function of cascading proteins. Therefore, depletion in any cascading protein of the Wnt/PCP pathway would likely result in nearly identical morphological defects. Recent studies have reported that double-outlet right ventricle, a major cardiovascular malformation, was found in mice with Dvl-2 or Daam-1 knockout, and 1 study reported mutation of the Daam-1 gene located on 14q23.1 in an aborted fetus with double-outlet right ventricle [45–49]. These findings suggest that Wnt-11, Dvl-2, and Daam-1 are a set of cascading proteins in the Wnt/PCP signal transduction pathway, which cannot be replaced by another signal transduction pathway.

A recent study using Xenopus embryos with Wnt-11 or Dvl-2 knockout reported that, after direct injection of the active form of Daam-1, the embryos exhibited dorsal formation, a function expected if the Wnt/PCP pathway is normal [50]. The Daam-1 used in this study was mutated to function independently without binary formation with activated Dvl-2 and was thus expected to compensate for the function of the Wnt/PCP pathway. These findings indicate that activated Daam-1 is capable of functioning and signal transduction even in the absence of activated upstream cascading proteins.

### Contribution of Daam-1 to IPAH pathogenesis

The present study revealed a characteristic Wnt/PCP signal transduction pattern in PASMC from IPAH patients. The positive expression rate was higher for downstream Daam-1 than for upstream Dvl-2; however, in PASMC from patients without pulmonary arterial abnormalities and APAH patients, the positive expression rate was lower for Daam-1 than for Dvl-2. These findings suggest that IPAH pathogenesis differs from that of APAH, although both diseases yield very similar pathological findings, ie, extreme hypertrophy of PASMC [13, 36, 47]. In addition, our finding of elevated Daam-1 expression in the presence of depleted Dvl-2 expression in medial SMC from IPAH cases suggests that Daam-1 is activated independently of Dvl-2. As mentioned above, the Wnt/PCP signal transduction pathway has no alternate collateral pathway; thus, a mutation of *Daam-1,* which is located on 14q23.1 [48–50], may subsequently activate the RhoA/ROCK pathway, resulting in aberrant medial SMC contraction and hypertrophy ultimately manifesting as IPAH [35]. Therefore, Dvl-2 underexpression in the context of Daam-1 overexpression in medial SMC from IPAH cases could be a result of a negative feedback mechanism in the signal transduction pathway. Indeed, at least 2 studies have reported negative feedback in the Wnt canonical and TGFβ superfamily pathways [31, 32].

## Limitations

The present study has 2 important limitations. First, the study was carried out on autopsy subjects that had received a diagnosis of IPAH or APAH. The autopsies were performed at Toho University Omori Medical Center during the period from 1958 through 2011. Because of the extremely low prevalence of PAH (2.4–15 cases per million), only a very small number of cases were identified: 9 IPAH cases and 7 APAH cases. Second, the entire analytical process was done by using IHC to confirm expressions of select cascading proteins of Wnt/PCP pathway, mainly because of technical difficulties. Other mechanistic methods such as real-time polymerase chain reaction, and gene ontology and pathway analysis based on DNA microarray assay technique assisted by laser microdissection, should be used in future studies.

## Conclusions

The presence of Daam-1 overexpression under conditions of Dvl-2 underexpression in medial SMC from IPAH patients suggests that unregulated upregulation of RhoA/ROCK results in aberrant medial SMC contraction and proliferation and, ultimately, IPAH. Further study of the mechanism of activated Daam-1 overexpression in the Wnt/PCP signal transduction pathway may shed light on IPAH pathogenesis.

## Abbreviations

APAH: Associated pulmonary arterial hypertension
BMPR2: Bone morphogenetic protein receptor type II
Daam-1: Dishevelled-associated activator of morphogenesis 1
Dvl-2: Dishevelled-2
FFPE: Formalin-fixed and paraffin-embedded
FPAH: Familial pulmonary arterial hypertension
HPAH: Heritable pulmonary arterial hypertension
IHC: Immunohistochemistry
IPAH: Idiopathic pulmonary arterial hypertension
JAK/STAT: Janus kinase/signal transducers and activators of the transcription
PA: Pulmonary arteries
PAH: Pulmonary arterial hypertension
PDGF: Platelet-derived growth factor
RhoA/ROCK: Ras homolog gene family, member A/Rho-associated, coiled-coil–containing protein kinase
SMC: Smooth muscle cells
TGFβ: Transforming growth factor β
VEGF: Vascular endothelial growth factor
Wnt/PCP: Wnt/planar cell polarity
